# Pre-metastatic niche: from revealing the molecular and cellular mechanisms to the clinical applications in breast cancer metastasis

**DOI:** 10.7150/thno.82700

**Published:** 2023-04-17

**Authors:** Yuqiu Li, Miao Li, Kangtai Su, Siwen Zong, Hongyan Zhang, Lixia Xiong

**Affiliations:** 1 Department of Pathophysiology, Medical College, Nanchang University, 461 Bayi Road, Nanchang 330006, China.; 2 Queen Mary College of Nanchang University, Nanchang 330006, China.; 3 Second Clinical Medical College, Nanchang University, Nanchang 330006, China.; 4 First Clinical Medical College, Nanchang University, Nanchang 330006, China.; 5 Department of Burn, The First Affiliated Hospital, Nanchang University, 17 Yongwaizheng Road, Nanschang 330066, China.

## Abstract

Breast cancer (BC) is one of the most commonly diagnosed cancers and the leading cause of cancer-related deaths in women worldwide. Metastasis is a major contributor to high cancer mortality and is usually the endpoint of a series of sequential and dynamic events. One of the critical events is forming a pre-metastatic niche (PMN) that occurs before macroscopic tumor cell invasion and provides a suitable environment for tumor cells to colonize and progress into metastases. Due to the unique characteristics of PMN in cancer metastasis, developing therapies to target PMN may bring new advantages in preventing cancer metastasis at an early stage. Various biological molecules, cells, and signaling pathways are altered in BC, regulating the functions of distinctive immune cells and stromal remodeling, inducing angiogenesis, and effect metabolic reprogramming and organotropism to promote PMN formation. In this review, we elucidate the multifaceted mechanisms contributing to the development of PMN in BC, discuss the characteristics of PMN, and highlight the significance of PMN in providing potential diagnostic and therapeutic strategies for BC metastasis, which may bring promising insights and foundations for future studies.

## Introduction

According to cancer data in 2023, breast cancer (BC) has overtaken lung cancer as the most common cancer in women worldwide 1. It is also the leading cause of cancer-related death, with metastases to vital organs remaining the leading cause of death in BC patients 2. Several studies have revealed that BC metastasis is heterogeneous, with different metastatic priorities in different organs. Bone, lung, liver, and brain are commonly considered to be the main targets of BC metastases 3. This organotropism leads to differentiation in prognosis and the effects of treatment in patients with BC. Therefore, it is important to identify pivotal molecular and cellular components in any cancer metastasis process and develop novel strategies for the early detection and treatment of BC patients.

The process of metastasis is complicated, and multiple molecules and events contribute to this process. One of the vital steps in cancer metastasis is the formation of a metastatic microenvironment 3. Proposed in 1889, Stephen Paget illustrated that metastasis depends on the interactions between "seed" (or cancer cells) and "soil" (or host microenvironment), opening new directions for understanding the metastatic process and the phenomenon of organotropism. Further research suggested that as the tumor progresses, a local supportive and receptive microenvironment called the "pre-metastatic niche" (PMN) forms to prepare tumor cells to colonize distant organs before reaching them, thereby promoting tumor settlement and metastasis. The formation of PMN is triggered and induced by PMN-promoting molecules (such as tumor-secreted factors, cytokines, chemokines, inflammatory factors, and exosomes), secreted by the primary tumor, the tumor-mobilized myeloid cells, and the local stromal cells of the host. With the intensive exploration of the PMN, the mechanism of the formation of the PMN in BC is gradually being understood. In recent years, research has shown that the PMN plays a critical role in cancer metastasis using various mechanisms, including immunosuppression, angiogenesis/vascular permeability, stromal remodeling, organotropism, and metabolic reprogramming 4. It has been found that BC cells form an osteogenic niche before osteolytic metastasis, and the proliferation capacity of BC bone metastasis is modified by stromal cells in PMN 5. In addition, the extracellular matrix (ECM) and immune cells, also as components of the PMN, contribute to the early colonization of BC in metastatic organs 6.

This review discusses different mechanisms for inducing PMN formation and shows how the PMN in BC allows disseminated cancer cells to survive and grow. Besides, we also elucidate the characteristics and significance of PMN in BC metastases, which can offer perspectives to identify new anti-metastatic targets and bring distinctive insights for preventing, early diagnosing, and treating BC metastasis.

## The formation of PMN

The unique concept of the PMN provides a prerequisite for organotropic metastasis in cancer cells. The PMN represents an aberrant, cancer-cell-free microenvironment that favors tumor cell growth, distinguishing it from the tumor microenvironment 7.

The formation of a PMN requires the interaction of three key factors: tumor-derived secreted factors (TDSFs), mobilization and recruitment of bone marrow-derived cells (BMDCs), and inflammatory polarization of stromal components in the tissue site 4. The PMN formation process is closely related to the process of cancer metastasis. It proves that the tumor metastasis process can be an ordered pathological event that can be divided into different stages in a temporal order. There are mainly four sequential stages 8. The first stage is the initiation phase and is remotely controlled by primary tumors. Primary tumors release soluble factors such as TDSFs, exosomes, and other molecules. These factors could trigger the formation of a non-maturing PMN at new organ tissue loci or non-orthotopic tumor sites in the same organ.

**Figure 1:**
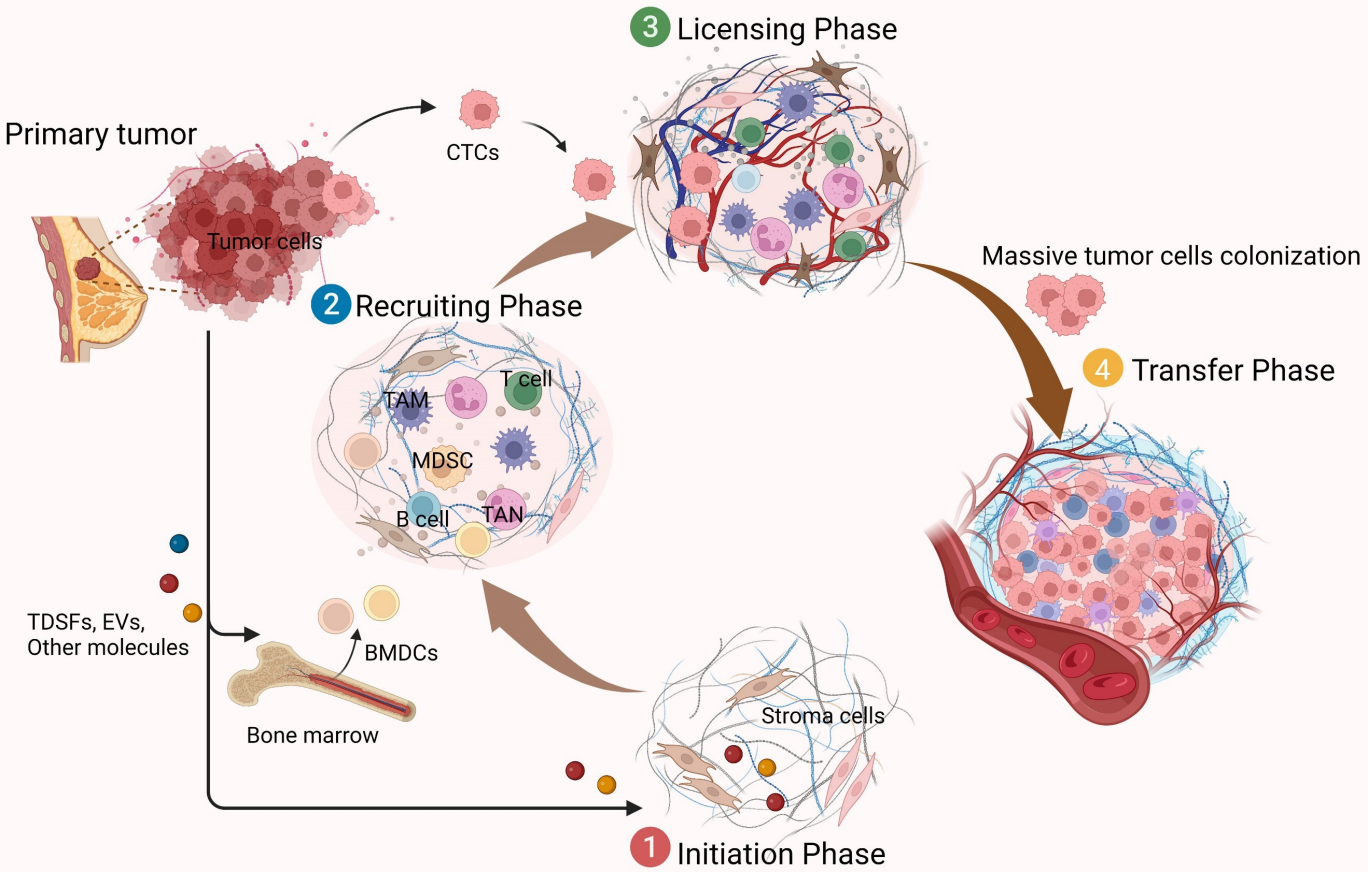
** Functions of the PMN in tumor metastasis.** The formation of PMN promotes tumor metastasis in several phases.①Initiation phase (remote control of primary tumors): Primary tumors produce soluble factors that induce the formation of a non-mature tumor pre-metastatic microenvironment; ②Recruiting phase: BMDCs and a variety of immunomodulatory cells are constantly recruited to specific sites, where they interact with stromal cells here to change the local microenvironment, forming a mature PMN; ③Licensing phase (preparation of the microenvironment): allow CTCs to reach and colonize; ④Metastatic phase: massive CTCs colonize and grow, and gradually form visible metastasis. The figure was created using Biorender (https://biorender.com/).

The PMN is not ready for cancer cell colonization at this point, but it is critical for the subsequent recruitment of BMDCs and regulatory immune cells, as well as for the formation and domestication of the host stromal environment. The second phase is the recruiting phase. Under the influence of TDSFs, BMDCs, and various immune cells are recruited to specific sites and interact with stromal cells to alter the local microenvironment. For example, induction of ECM remodeling and activation of integrins and chemokines may form a mature niche conducive to tumor cell colonization. Subsequently, in the licensing phase, the distally preformed PMN allows circulating tumor cells (CTCs) to reach and colonize. Some of these CTCs survive and form micrometastases, while others become dormant (until the microenvironment is suitable for tumor growth). Eventually, when the metastatic phase occurs, they form clinically visible metastatic lesions as the number of recruited and colonized tumor cells increases **(Figure 1)**. In these dynamic processes, the specific properties of PMN are considered critical for promoting the effective colonization of tumor cells and the growth of de novo metastatic tumors 9. In addition to TDSFs, exosomes and other molecules, including local hypoxia, promote tumor PMN formation. It has been found that hypoxia-inducible factor-1α (HIF-1α) stabilization by hypoxia and transforming growth factor-β (TGF-β) increases expression of the ECM-modifying enzyme collagen prolyl-4-hydroxylase in BC cells 10, 11. Thus, high-stability collagen is deposited in the lung PMN, which supports metastatic growth and inhibits prolyl-4-hydroxylase, thereby affecting the lung PMN in BC formation.

## Mechanisms implicated in PMN in BC metastasis

**Figure 2:**
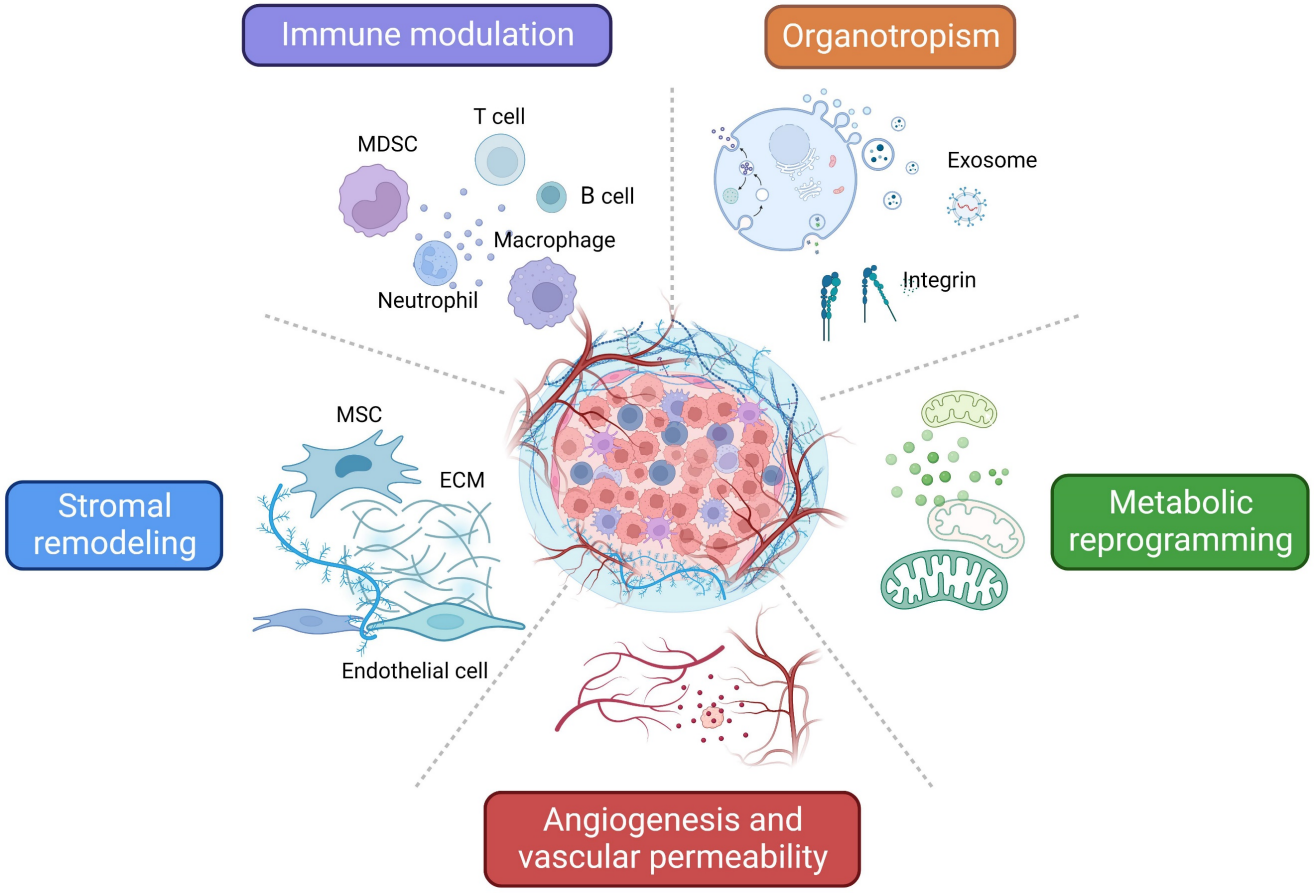
** Characteristics and related mechanisms of PMN in BC.** An overview of the characteristics and related mechanisms of PMN in BC can be summarized as follows: regulation of immune cells, stromal remodeling, angiogenesis and vascular permeability, metabolic reprogramming, and organotropism. The figure was created using Biorender (https://biorender.com/).

As mentioned above, TDSFs, exosomes, and other molecules derived from primary tumors induce dynamic changes that promote PMN formation and ultimately elicit critical roles during BC early metastasis. Here we summarize the different mechanisms and characteristics of PMN related to BC metastasis **(Figure 2).**

### Regulation of immune cells

As a vital link in tumor metastasis, the immune system elicits important functions in PMN formation and metastatic colonization of tumor cells. Several researchers have found that various types of immune cells are extensively involved in PMN formation in BC and promote effective colonization of tumor cells 12. The key factors in PMN formation are the recruitment of various immune cells, including myeloid-derived suppressor cells (MDSCs), neutrophils, macrophages, and regulatory T/B lymphocytes to the target organs and the performance of their roles 4, 9.

**Figure 3:**
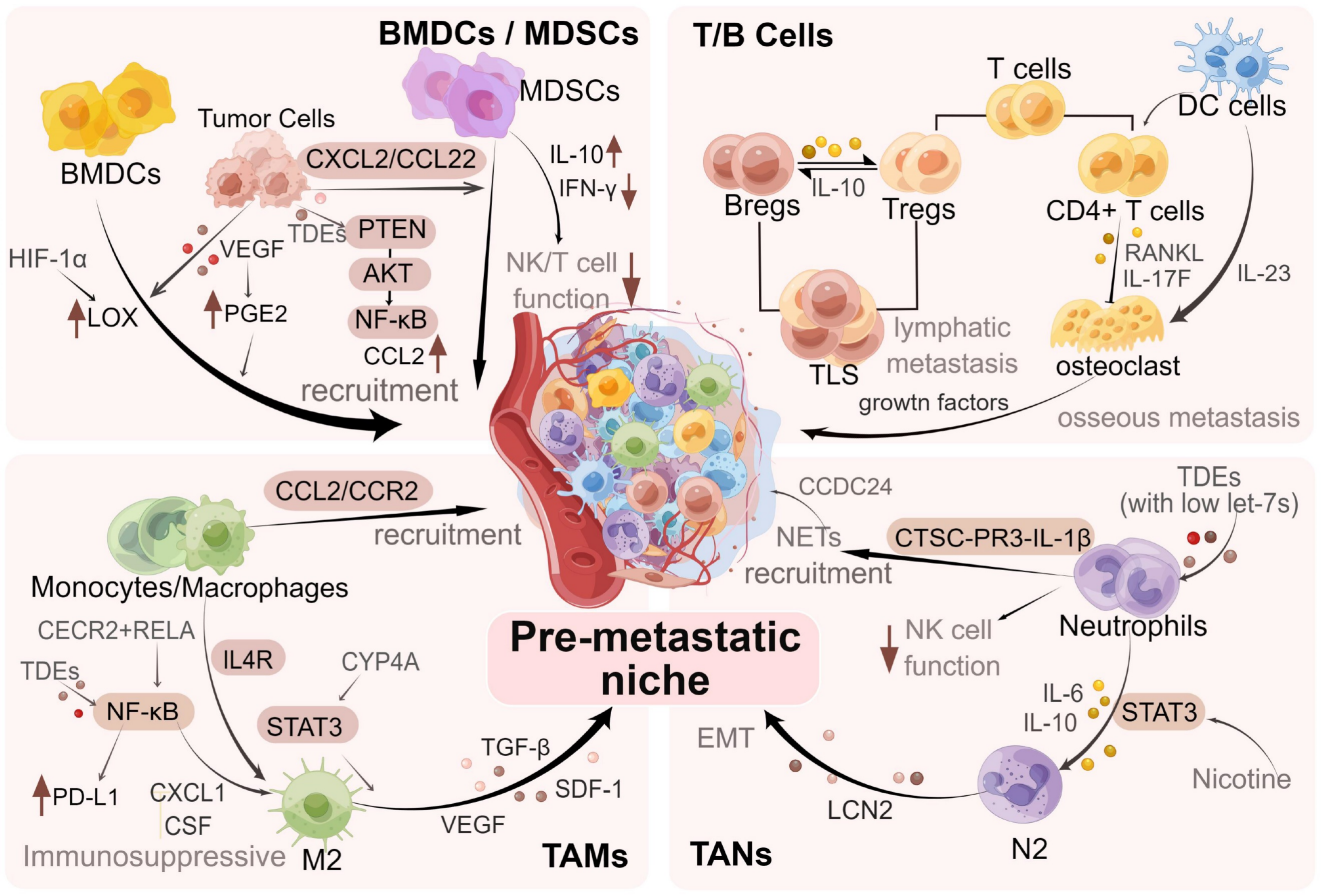
** Regulation of immune cells in PMN in BC.** During the formation of PMN in BC, immune cells such as MDSCs, neutrophils, macrophages, and regulatory T/B lymphocytes are recruited, regulated and modified via different mechanisms and signaling pathways, which promote the colonization of tumor cells and further metastasis. The figure was created by Figdraw (https://www.figdraw.com/).

For example, macrophages expressing vascular endothelial growth factor receptor-1 (VEGFR-1) are recruited into the BC lung PMN 13. Bone marrow-derived (Gr-1^+^) cells have been illustrated to prove liver metastases in BC 14. Furthermore, it has been found that neutrophils can serve as important mediators to activate dormant tumor cells in the lung PMN 15. The above studies suggest the value of interactions between cancer cells and immune cells in the PMN, indicating the significance of revealing the underlying mechanisms. We specifically discussed the roles of MDSCs, T and B cells, macrophages, and neutrophils in promoting PMN formation and BC metastasis **(Figure 3)**.

#### Recruitment of MDSCs

BMDCs are major participants in PMN formation and one of the key cellular components of PMN 16. Extracellular signals released from primary tumors are crucial in altering the microenvironment at distant organ sites. This could induce the recruitment of BMDCs to pre-metastatic sites before cancer cells arrive, thereby promoting PMN formation. For example, vascular endothelial growth factor (VEGF) secreted by 4T1 breast cancer cells can induce prostaglandin E2 (PGE2) expression to promote homing of BMDCs to the lungs before tumor cells arrive 17. Joo and Wong et al. found that primary tumor cells recruited BMDCs to metastatic sites through the HIF-1α/lysyl oxidase (LOX) axis to promote PMN formation 18, 19.

However, as a heterogeneous population derived from bone marrow, MDSCs are immature bone marrow cells that can appear in early PMN and have potent immunosuppressive properties 20. MDSCs are mainly classified as subgroups of mononuclear MDSC (M-MDSC) and granulocyte MDSC (G-MDSC) (or polymorphonuclear MDSC), which have different immunosuppressive mechanisms 21. Sceneay et al. illustrated that hypoxia-driven immunosuppression could regulate PMN 22. Hypoxic primary BC cells produce TDSFs to promote the recruitment of G-MDSCs (granulocyte CD11b^+^/Ly6C^med^/Ly6G^+^ subset) to the lungs that, in turn, suppress NK-cell function 22. It has been demonstrated that the attraction of MDSCs to the pre-metastatic site can also establish an immunosuppressive niche by regulating the production of immunosuppressive cytokines such as IL-10, which suppress antigen-specific and non-specific T cell responses 20, 23. Immature Gr-1^+^ CD11b^+^ myeloid cells in lung PMN could decrease the production of IFN-γ and increase Th2 cytokines to reduce host immune surveillance 24. Therefore, suppression of immune cell responses could be an important criterion for defining MDSCs.

In addition, it has been revealed that BC cell-derived exosomal miRNA-200b-3p could target the 3'-UTR of tumor suppressor-phosphatase and Tensin homolog (PTEN) and inhibit their synthesis, activating the protein kinase B (AKT)/nuclear factor kappa-B (NF-κB) pathway and leading to the secretion of the C-C patterned chemokine ligand 2 (CCL2) 25. Due to the high expression of CCL2 in the lungs, MDSCs are recruited and mediate the construction of the lung PMN 25. Another study also found that CCL2 increased the migration of MDSCs and induced the secretion of S100A8/A9 (an exosome protein that contributes to metastasis, angiogenesis, and immunosuppression), creating a tumor-permissive, immunosuppressive environment in metastatic target organs 26. MDSCs were abundant in lung PMN and associated with subsequent metastatic BC burden through S100A8/A9 imaging 26. Therefore, it is speculated that MDSCs play important roles in BC lung metastases, but the specific mechanism deserves further investigation. In conclusion, MDSCs in PMN can achieve their immunosuppressive characteristics through multiple pathways to establish an immunosuppressive environment for subsequent colonization of tumor cells.

Besides, MDSCs are also involved in several non-immune functions that may be related to the PMN through the secretion of cytokines, chemokines, and growth factors. Several studies have reported the presence of higher levels of ΔNp63 (an isoform of p63) in triple-negative breast cancer (TNBC) and abundant polymorphonuclear MDSCs in the metastatic microenvironment of tumors 27. Highly expressed ΔNp63 promoted the recruitment of MDSCs through transcriptional activating chemokines (CXCL2/CCL22) and induced their pro-tumorigenic factor secretion, such as chitinase 3-like 1 (CHI3L1) and MMP9, thereby enhancing the metastatic potential of TNBC 27. A deficiency of ΔNp63 also inhibited angiogenesis in TNBC 27. Furthermore, both human and mouse TNBC cells confirmed the pro-metastatic role of MDSCs in normal and impaired immunity models, highlighting the non-immunological role of MDSCs in TNBC. This might suggest that MDSCs may contribute to metastasis by promoting PMN formation, angiogenesis, and tumor cell invasion.

Overall, the roles of MDSCs in the formation and evolution of PMN are diverse, including establishing a favorable immunosuppressive environment before metastasis, promoting angiogenesis, and enhancing tumor cell invasion 28. The intrinsic relationship between tumor cells and recruitment of MDSCs and the multiple roles of MDSCs in BC metastasis still need to be explored, which may be of seminal importance for the combined treatment of BC.

#### Regulation of T or B cells and functions

The role of immune-specific T or B cells in tumor proliferation and metastasis has been extensively studied. Activation of T or B cells is mediated by the presentation of antigen-presenting cells (APCs), such as dendritic cells (DCs) circulating in the peripheral blood, and may support tumor metastasis to distant organs 29.

Studies have shown that 70% of patients with advanced BC develop bone metastases, significantly increasing BC mortality. This is due to osteoclasts (OCs)-driven osteolytic lesions in the bone marrow (BM) microenvironment, which promote metastasis of BC to bone 30. Studies have also demonstrated that tumor-specific T cells alter the bone microenvironment before bone colonization in BC bone metastasis 31. The specific T cells are typical RANKL+ IL-17F+ CD4+ T cells. These cells arrive in the bone marrow before the tumor cells and establish a PMN that stimulates bone depletion, causing the release of growth factors stored in the mineralized matrix to provide nutrients for the tumor, creating a vicious cycle between the bone remodeling system and the tumor 30, 32.

Monteiro et al. showed that the pro-metastatic activity of T cells is correlated with the receptor activator of nuclear factor kappa B ligand (RANKL), a key regulator of osteoclastogenesis 31. However, the 67NR^+^ tumor-derived CD8^+^ T cells highly expressed IFN-γand IL-10 and produced low levels of RANKL 32. This environment inhibits the 4T1 tumor-specific CD4^+^ T cell phenotype and disrupts osteoblast and osteoclast activity in the BM 32. This suggests an opposing role of CD4^+^ and CD8^+^ T cells in regulating BC progression and bone metastasis.

DCs and T cells are the primary T cell partners in the initial phase of the immune response and have come under the spotlight as a potential source of progenitor cells of OCs in inflammatory conditions 30. DCs can differentiate into efficient bone-resorptive osteoclast-like cells that express IL-23 cytokines and retain their APC properties to maintain the pro-osteoclastogenic T cell phenotype in the BM 30, 32. This suggests that DCs provide a positive feedback loop for metastatic tumor-induced osteolytic diseases.

Furthermore, one study found the immune crosstalk between tumor-infiltrating B cells (TIL-Bs) in lymph nodes (LNs) and BC, mediating the formation of a pre-lymph node metastatic niche 33. Tumor-infiltrating B cells could modulate the immune response of tumors by binding to T cells to form tertiary immune structures (similar to the germinal centers in the LNs) 33. Previous studies have demonstrated that tolerance to germinal centers (GC) response is partially maintained by follicular regulatory T cells (Tfr), circulating Tfr, and regulatory T cells (Tregs). These cells are increased in BC patients, particularly in cancers with more aggressive abilities 34, 35. Tfrs and Tregs can induce the immunosuppressive microenvironment and promote the expansion of IL-10-producing B cells (immunosuppressive-producing B cells, so-called Bregs) in BC 36. Increased IL-10-secreting Bregs, in turn, enhance the function of Tregs 37. Since the cells and morphology of tertiary lymphoid structures (TLS) are similar to those of GC, we speculated that there might be mechanical similarities between these two structures. In BC LNs metastasis, TIL-Bs can depend on IL-10 and cooperate with Tregs to construct an immunosuppressive microenvironment.

Overall, T/B cells play a major role in establishing immunosuppressive PMN in BC, and there is an interaction between the two. However, T cells with different phenotypes have different directions of immunomodulatory functions and have dual roles.

#### Infiltration and polarization of macrophages

As a key component of the tumor microenvironment, macrophages promote tumor development and metastasis. Infiltration at metastatic sites through stimulation of monocytes/macrophages also contributes to PMN formation 38. Research has shown that macrophages could regulate BC transmission and metastasis 39. It has been found that tumor-derived exosomes may be involved in the induction of macrophage polarization 40. It can polarize macrophages to an immunosuppressive phenotype in PMN through NF-κB-dependent glycolytic metabolism, increase programmed cell death-ligand 1 (PD-L1) expression, and then exert an immunosuppressive effect 40.

Gong et al. showed that a population of pre-pulmonary fibroblasts expressing cyclooxygenase-2 (COX-2) produces PGE2 to drive dysfunctional DCs and inhibitory monocytes, thereby reshaping the immune microenvironment in the lungs 41. It can also support pulmonary PMN by transmitting the pro-inflammatory cytokine interleukin-1β 41. Also, one study found that it drives the monocyte/macrophage responses via the CCL2/CCR2 axis, thus recruiting macrophages to PMN to promote lung colonization of BC cells 42. These studies confirmed that activating β-adrenergic signaling under chronic stimulation significantly enhanced CCL expression in lung tissues, inducing macrophage infiltration 42. Similarly, in BC bone metastases, monocyte/macrophage depletion significantly inhibited the growth of bone metastases 43. Ma et al. showed that macrophages promoting bone metastases are mainly associated with CCR2-dependent recruitment of inflammatory monocytes instead of tissue-resident macrophages 43. This is due to the high expression of IL4R on recruited bone metastasis-associated macrophages (BoMAMs) in the monocyte-derived macrophage population, where IL4R signaling is essential for polarization and the native function of BoMAMs 43.

Furthermore, most tumor-associated macrophages (TAMs) mainly exhibit a M2-like pro-tumor phenotype, and their significance in metastasis has been widely elucidated 44. Emerging evidence shows that metastases in distant BC have increased M2 macrophage numbers and are more immune-indolent than their matched primary tumors 45. It indicates that certain epigenetic aberrations drive tumor growth and metastasis by activating genes promoting metastasis and creating immunosuppressive microenvironments, such as cat eye syndrome chromosome region candidate 2 (CECR2) 45. It has been experimentally demonstrated that CECR 2 activates NF-κB target genes, including the cytokine genes CSF1/2/3, CXCL1, and various pro-metastasis genes, through its interaction with the acetylated v-rel avian reticuloendotheliosis viral oncogene homolog A (RELA) that recruit and induce M2 polarization and proliferation of M2 TAMs, creating an immunosuppressive microenvironment 45. In addition, Chen et al. found that cytochrome P450 (CYP) 4A in the TAM could induce macrophage M2 polarization via the signal transducer and activator of transcription 3 (STAT3) signaling 46. The authors also demonstrated that promoting the production of M2 macrophage-derived cytokines (TGF-β, SDF-1, and VEGF) induced PMN formation 46.

The above studies indicate that different macrophage subsets may play a dominant role in the progression of BC and different metastatic organs. Macrophage heterogeneity is an important component of the PMN that determines the tissue-specific metastasis mechanism. Therefore, further studies on the different subtypes and functions of macrophages are needed to develop a precise BC-targeted therapy.

#### Infiltration and polarization of neutrophils

Studies have shown that a high neutrophil-to-lymphocyte ratio is closely related to poor prognosis in BC metastatic 47. One study revealed that oxysterol in BC cells supported the metastatic cascade by altering the lung PMN, specifically allowing the recruitment of tumor-promoting neutrophils 48. This indicates the importance of neutrophils in BC metastases.

Like macrophages, neutrophils could also polarize the N1 or N2 phenotype and play different roles in tumor progression 49. Among them, neutrophil N2 transformation is a key factor that inhibits T cell function. Qi et al. demonstrated that Lin28B expression induces neutrophil recruitment and N2 transformation to establish immunosuppressive PMN 50. This is mediated by the involvement of tumor-derived exosomes (TDEs) with low let-7s that mediate the production of cytokines (IL-6, IL-10) and induce N2 transformation 50. In addition, neutrophil polarization is also dependent on the STAT3 signaling pathway. Tyagi et al. showed that neutrophil N2 transformation was induced by STAT3 signaling after nicotine stimulation to establish PMN in the lungs 51. Besides, nicotine stimulation also induced the mesenchymal-epithelial transformation of tumor cells by promoting the release of apolipoprotein 2, thereby promoting colonization and metastatic growth 51.

In addition, neutrophils could create an inflammatory microenvironment that facilitates BC lung metastasis. Another mechanism of PMN formation related to neutrophils is neutrophil extracellular traps (NETs), a germicidal mechanism of neutrophils that eliminates bacteria by emptying their chromatin content into the extracellular space. Nuclear chromatin, related nuclear histones, and cytoplasmic and granular antimicrobial proteins make up the structures of NETs 52. One study showed BC NETs could form before lung metastasis by activating the C3-C3aR axis and attracting neutrophils regulated by the STAT6 pathway. 53. NETs have critical roles in cancer metastasis. By binding to CCDC24, the chromatin DNA component in NETs is considered a chemotactic factor for tumor cell recruitment 54. The NETs-related proteases can split the laminin protein, one of the ECM components, to cause laminin remodeling and regulate integrin-mediated signaling in tumor cells, promoting tumor cell proliferation 15. The accumulation of NETs promotes the establishment of lung PMN, in which more tumor cells are trapped and colonized in the lung. Therefore, BC is more likely to metastasize to lung tissues 53.

Xiao et al. revealed the functions of the tumor-secreted protease cathepsin C (CTSC) in promoting neutrophil recruitment and NET formation 55. More recently, it has been demonstrated that CTSC can activate neutrophil membrane-bound proteinase 3 (PR3) to promote the activation of nuclear factor-kB and regulate interleukin-1b (IL-1b) to increase IL-6 and CCL3. The production of neutrophil reactive oxygen species and the formation of NETs are induced by the CTSC-PR3-IL-1b axis, which degrades thrombospondin-1 and supports lung colonization of breast cancer 55.

However, the functions of neutrophils in cancer metastasis are complicated. Recent investigations have demonstrated that neutrophils, natural killer cells (NK), and tumor cells interact 56. NK cells have a stronger tumor-killing effect than neutrophils. On the other hand, neutrophils inhibit NK cells and elicit cytotoxic effects on tumor cells. Therefore, neutrophils without NK cells only showed tumor-killing ability or net anti-metastatic effects. In contrast, in the presence of NK cells, neutrophils inhibit the killing of more NK cells, resulting in a net pro-metastatic effect. The dual role of neutrophils depends on the ROS signaling pathway 56.

### Stromal remodeling

The stromal microenvironment, comprising fibroblasts, pericytes, mesenchymal stem cells (MSCs), endothelial cells, ECM, and vasculature, is crucial for the survival of cancer cells at the metastatic sites 57. During PMN formation in BC metastases, remodeled ECM and supportive signals from stromal cells are important for tumor colonization and metastasis 58.

#### Onco-Promoting effects elicited by MSCs

Mesenchymal stromal cells (MSCs) are a group of heterogeneous cells located mainly in the BM with distinct abilities and plasticity that can regenerate and maintain diverse connective tissues, including bone, cartilage, adipose, and muscle 59. In addition, various bone marrow stromal cells such as fibroblasts, endothelial cells, pericytes, osteoblasts/osteocytes, adipocytes, and chondrocytes are derived from MSCs, which are involved in developing PMN and facilitating cancer metastasis 60. Although MSCs are a small cellular portion of the bone marrow, they are vital in hematopoietic homeostasis since they regulate immunological functions and osteoclastogenesis processes 61, 62.

Systematic reviews of the available data revealed that MSCs are attracted to microenvironments such as inflammatory tissues rich in growth factors, cytokines, and chemokines 63. MSCs can be recruited by primary BC, which subsequently affects the metastatic potential of BC 64. Studies have revealed that factors and chemokines such as IL-6, CCL-2, CCL-5, and hypoxic situations in the BC microenvironment can attract MSCs and promote IL-6 release by BC cells 65-67. After recruitment to the tumor microenvironments, MSCs are activated and transformed into tumor-associated MSCs 67. The activated pro-tumor MSCs can increase the production of soluble factors, including CCL-2 and TGF-β that regulate the immune responses in the primary tumor and facilitate the recruitment of monocytes and MDSCs to create the immunosuppressive tumor microenvironment 68, 69. Besides, tumor-associated MSCs can also become cancer-associated fibroblasts (CAFs). The extensive evidence in the literature shows that CAFs in BC could release several cytokines and growth factors that promote tumor growth and angiogenesis 70. Some literature revealed that MSCs could induce the epithelial-mesenchymal transition of tumor cells by releasing multiple factors such as TGF-β, basic FGF, hepatocyte growth factor (HGF), and epidermal growth factors related to stemness 71, 72.

In BC bone metastases, many studies have indicated that MSCs may elicit critical functions in promoting BM/bone PMN formation 73. After comparing the peripheral blood plasma of untreated advanced BC patients (clinical-pathological stage III-B, without BM/bone metastases) with healthy subjects, Martinez et al. found that the ability to increase the transendothelial migration of BC cells was increased in peripheral blood and bone marrow plasma in BC patients 74.

The high concentrations of RANKL, macrophage migration inhibitory factor (MIF), and osteoprotegerin (OPG) in the peripheral blood of patients may contribute to the process of intravasation, angiogenesis, survival, and epithelial-mesenchymal transition of circulating tumor cells 74. Levels of PDGFAB, ICAM-1, and VCAM-1, considered vital components for recruiting pre-osteoclasts, promoting osteoclastogenesis and BM extravasation, were also elevated in the bone marrow of patients. These findings indicate their roles in facilitating the proliferation and extravasation of cancer cells and bone resorption 74. During these processes, MSCs can enhance bone resorption by decreasing OPG production, which can bind to soluble RANKL and regulate the recruitment of tumor cells and modify the migration of BC cells 74. Collectively, this literature suggested that changes existed in BM and that the MSCs were involved in these processes in untreated BC patients, contributing to the formation of PMN and BC bone metastases.

Upon interaction with immune cells and immune factors, tumor MSCs can secrete various growth factors and chemokines to promote cancer development and metastasis. The evidence to date suggests that local MSCs can attract monocytes, macrophages, and neutrophils to establish the tumor immune microenvironment that promotes PMN formation and cancer progression 67, 75, 76. Zheng et al. illustrated that before BC lung metastasis, lung MSCs are stimulated by Th2 cytokines such as IL-4/IL-13 via the STAT6 pathway to release high levels of complement component 3 (C3), thereby attracting neutrophils to the lungs through the C3-C3aR axis and form the NETs 53. As mentioned above, NET facilitated the cleavage of proteins of ECM components and regulated the pathways of tumor cells, promoting the establishment of lung PMN and was conducive to BC lung metastasis 53.

#### Activation of endothelial cells

During cancer metastasis, normal quiescent endothelial cells that previously acted as a barrier can be activated by pro-inflammatory mediators to express more pro-inflammatory adhesion molecules and release more pro-inflammatory cytokines that facilitate the recruitment of tumor cells or bone marrow-derived hematopoietic cells 77-79. For instance, the upregulation of endothelial adhesion molecules such as E-selectin benefits cancer metastasis by promoting the rolling and arrest of BMDCs and tumor cells 80-82. An increase in cytokines like IL-8 result in inflammation, recruit pro-inflammatory cells and promote the secretion of growth factors and chemokines to contribute to cancer metastasis 83. Hence, it can become one of the first steps involved in PMN formation. Furthermore, previous studies suggested that TDEs might also activate endothelial cells 84. Che et al. showed that tissue factor (TF) expressing BC-derived exosomes could activate quiescent endothelial cells, thereby upregulating adhesion molecules and cytokines. This activation is regulated by the TF-dependent production of FXa and activation of protease-activated receptor 1 (PAR-1). Switching from quiescent endothelial cells to the activated phenotype promotes BC metastasis and PMN formation 85.

#### Alteration of extracellular matrix dynamics

The extracellular matrix (ECM) is a dynamic extracellular environment that constantly degrades, accumulates, and changes 86. Studies have shown that aberrant ECM deposition and remodeling like proteolysis and crosslinking may irreversibly change the microenvironment and transform it into a pro-tumor microenvironment, which is thought to be the early change that establishes the polymorphonuclear neutrophils (PMN) 87. Several ECM components, such as collagens, hyaluronan (HA), Fibronectin (FN), and Tenascin-C (TNC) play important roles in early PMN and promote breast cancer metastasis 6.

Mesenchymal stem cells (MSCs) derived Hyaluronan (HA) can regulate the properties of PMN. Hyaluronan surrounds MSCs and binds to its receptor CD44 to modulate MSC plasticity, changing the cellular composition and cellular interaction of the metastatic niche 88. Vogeley et al. investigated the highly invasive BC cells MDA-MB-231 and glioblastoma multiforme cells U87-MG to demonstrate the pro-tumor effects of HA on bone marrow MSCs and their interactions with tumor cells 89. Their findings showed that MDA-MB-231 cells inhibit the production of HA in MSCs, and this causes MSC adipogenic differentiation to be dysregulated. Therefore, it preserves the pro-tumor effects of MSCs and promotes tumor growth. Besides, the HA-rich matrix generated by BC cells and the HA receptor LAYN can enhance the adhesion of MDA-MB-231 cells to MSC. This way, BC cells regulated the PMN formation by changing the properties of the MSC HA-matrix 89. Collagens, as the main ECM proteins, provide structural support and bind to other ECM proteins. As tumors develop, hypoxia in the tumor microenvironment occurs frequently, resulting in the production of lipoxygenases (LOX). Many studies have shown that BC expresses LOX to crosslink collagen IV fibers in distant tissues, which enhances the adhesion of CD11b^+^ BMDCs and PMN formation 90. According to Kaplan et al. the primary tumors produce factors that cause the deposition of ECM components such as FN in the lungs, resulting in the adhesion of VLA-4^+^ VEGFR1^+^ hematopoietic progenitor cells (HPCs) in the lungs 91. Consequently, a favorable microenvironment for disseminated tumor cells (DTC) to seed is created and colonized 91. The Tenascin-C (TNC)expressed by BC cells promotes the colonization of new DTC to lung tissues by regulating the *Wnt* and *Notch* signaling. Increased TNC levels are also being investigated in the stromal cells and with the activation of myofibroblasts that promote the BC macro-metastases 92.

### Angiogenesis and vascular permeability

During tumor metastasis, tumor cells enter the bloodstream and travel to remote target tissues where they extravagate, invade the matrix, and effectively colonize and multiply 93. The completion of this metastatic process is dependent not only on the tumor cells' features but also on the tumor-promoting changes of the microenvironment at the location of distal metastasis 7.

Previously, the factors that regulate PMN were extensively reviewed 4, 7. It is been reported that establishing a metastatic microenvironment with high levels of pro-angiogenic factors (such as vascular endothelial growth factor (VEGF) in the PMN can activate an angiogenic switch and facilitate later cancer metastasis 94. Liu et, al. discovered that after subcutaneously injecting 4T1 BC cells into the mammary glands of mice, the expression of VEGF increased and the production of prostaglandin E2 (PGE2) was induced in the microvascular endothelial cells of the lung tissues of mouse, which enhance vascular hyperpermeability, upregulate endothelial adhesion molecules, and tumor cells homing 17. Alternatively, the induced increase in PGE2 could serve as a chemoattractant to promote the mobilization of bone marrow-derived cells (BMDCs) before the arrival of tumor cells and allow their recruitment to the lungs to promote tumor cell colonization. This also suggests that in addition to promoting vascular changes, VEGF may play a role in creating other potential PMN mechanisms.

Moreover, as new agents of tumorigenesis and tissue-specific metastasis, tumor‐derived exosomes (TDEs) are involved in cancer progression by transporting many angiogenesis-promoting biomolecules such as VEGF, matrix metalloproteinases, and miRNAs 95. MiRNA-105 can be found in circulation during the pre-metastatic period of early BC 84. By targeting the tight junction protein ZO-1 released by the tumor cells, miRNA-105 or the exogenous exosomal miRNA-105 can change vascular permeability in distant tissues and induce metastasis 84. Furthermore, Sayantan et al. demonstrated that exosomal Annexin II promotes angiogenesis and initiates the PMN formation in BC lung and brain metastases through the p38, NF-ĸB, and STAT3 pathways 96.

Furthermore, Lee et al. discovered that tumor-conditioned lymphatic endothelial cells (LECs) have a pro-angiogenic effect 97. Under normal circumstances, pro-angiogenic factors secreted by LECs are insufficient to induce angiogenesis. However, LECs under tumor conditions exhibit an angiogenic phenotype and promote angiogenesis in BC LNs, enabling tumor extravasation and colonization 97. Tumor-secreted IL-6 causes STAT3 phosphorylation in LECs within pre-metastatic LNs, which induces VEGF expression in LECs and promotes angiogenesis in LNs 97. Li et al. found that CD4^+^ T cells, CD8^+^ T cells, and B cells upregulated angiogenic pathway genes in tumor-draining lymph nodes (TDLNs) 98. This suggests that immune cells may be involved in causing increased angiogenesis in TDLNs.

In a nutshell, greater angiogenesis and vascular permeability in the PMN promote tumor cell invasion and development.

### Metabolic reprogramming

Metabolic reprogramming, considered one of the vital features of PMN, contributes to the niche for cancer cell colonization and metastasis. Metastatic tumor cells are well known to require a pattern of energy and nutrient metabolism to compete with resident cells in the ecological niche, adapt to the microenvironment of local tissues, and create an environment suitable for metastasis. In a rat model of BC, fibroblast reticular cells in TDLNs were found to have higher OXPHOS activity 98. This shows that cytokines produced by tumor cells may coordinate the metabolic reprogramming of the cells in various physiological or pathological conditions, thus promoting cancer metastasis.

TDEs have been shown to regulate cell metabolism within specific organs and aid in the formation of PMN. For instance, TDEs have been found to regulate hydroxyacid oxidase 1 (HAO1)-mediated oxalate metabolism in BC lung metastasis 99. BC cells activate TLR3-IRF3 signaling by producing exosomes, which induces HAO1 expression, and oxalate accumulation in alveolar epithelial cells. Moreover, oxalate accumulation in the lung induces NET formation by activating nicotinamide adenine dinucleotide phosphate oxidase, thereby promoting the establishment of PMN. They also discovered that oxalate enhances the proliferation of metastatic cancer cells by activating MAPK signaling 99. Another study found that tumor-secreted miRNA-122 changes the metabolism of lung and brain resident cells by downregulating the glycolytic enzyme pyruvate kinase (PKM) to increase glucose availability in PMN 100. Furthermore, more studies have shown that neutral lipids collect in lung-resident mesenchymal cells during the pre-metastatic stage and can be transported to tumor cells and NK cells by exosomes. This leads to higher tumor cell survival and proliferation, as well as dysfunction of NK cells, hence favoring BC lung metastasis 101. In addition to stromal cells in the PMN, certain immune cell activities appear to be closely related to the regulation of metabolic reprogramming. A recent study discovered that TDEs stimulate macrophage PD-L1 expression in PMN via glycolysis-dominated metabolic reprogramming 40. TDEs elevated nitric oxide synthase 2, which inhibits mitochondrial oxidative phosphorylation and increases the conversion of pyruvate to lactate. However, the increased lactate-feedback NF-κB signaling pathway further increased PD-L1 expression.

Metabolic reprogramming not only elicits vital functions in cancer-associated surrounding tissues, but it also plays key roles in shaping the PMN in BC metastasis. Huang et al. revealed that BC changes the lipid metabolism of lung fibroblasts by downregulating the expression of ACACA (acetyl-CoA carboxylase α), causing lung fibroblasts to become senescent and inflamed 102. Moreover, this senescence-associated secretory phenotype of lung fibroblasts may increase the secretion of CXCL1, which further promotes the recruitment of immunosuppressive G-MDSC to create an immunosuppressive lung PMN in BC 102.

Additionally, since the nutrients are available in different organs, cancer cells adjust the activity of their metabolic pathways to accommodate the nutrients in PMN 103. For example, BC cells in the lungs catabolize proline to meet their energy requirements and depend on pyruvate to shape the PMN 104, 105. However, pyruvate is a nutrient particularly abundant in the lungs. Thus, this could be one of the factors influencing the organotropism of metastatic cancer cells.

Although the detailed mechanisms of metabolic reprogramming in the formation of PMN in BC are unknown, these limited studies have shown the significance of metabolic reprogramming in the formation and progression of PMN, including changed lipid metabolism, altered glucose metabolism, and a series of enzymes, substrates, and signaling pathways, which provides a new perspective for further exploration of distant metastasis of BC.

### Organotropism

Organotropism is the process in which various kinds of cancer have varying propensities to metastasize to different organs. Many studies have indicated that PMN formation in particular organs leads to the organotropic metastasis pattern, which trains and transforms the target organ into a microenvironment suitable for the primary tumor to seed and colonize 9. The most common metastatic locations for BC are bone, lung, liver, and brain 106.

Tumor-derived factors and exosomes play important roles in the organotropic formation of PMN. TDEs transport different cargos such as microRNA and proteins, from main tumors to the secondary metastatic sites, causing changes in the local stromal cells. It was previously demonstrated that in BC lung metastasis, miR-200b-3p transported by BC-derived exosomes was taken up by alveolar epithelial type II cells, and MDSCs were recruited to the lung to form PMN through the PTEN/AKT/NF-κB/CCL2 cascade and promote the spread of BC cells to the lungs 25. Furthermore, Feng et al. used high-throughput sequencing to propose that BC-derived exosomes may promote lung fibroblast proliferation and migration by dysregulating the expression of many LncRNAs, which facilitates lung PMN formation 107. Additionally, primary TDSFs could activate the p38α kinase in lung fibroblasts, inactivating the type I interferon signaling and stimulating the production of fibroblast activation protein (FAP). The expression of FAP is required for ECM remodeling, including the accumulation of FN and the release of chemokines to attract neutrophils, which infiltrate lung tissues and facilitate the established lung PMN and further lung metastasis 108. In BC bone metastasis, BC-derived exosomes transport miR-21 to osteoclasts to promote differentiation and activation by decreasing the expression level of programmed cell death 4 (PDCD4), which remodels the microenvironment and accelerates the formation of bone PMN for bone metastasis 109. Furthermore, the different integrin expression profiles contribute to the organotropic establishment of the PMN that is induced by tumor-derived exosomes. Exosomal integrins α6β4 and α6β1 have been linked to lung metastasis, while αvβ5 contributes to liver metastasis 79. Additionally, although TDEs have been shown to promote PMN formation, their secretion and ability to form PMN are under control. Ghoroghi et al. discovered that the Ral family GTPases regulate the biogenesis and secretion of pro-metastatic exosomes via the phospholipase D1. On the other hand, RalA and RalB decrease the amounts of adhesion molecules in exosomes to promote the metastatic ability of exosomes targeting the lung, whereas exosomes from RalA or RalB deficient cells have fewer organotropic abilities 110.

TDEs may be created and changed as potential therapeutic targets for treating the development of PMN in various distal organs and BC metastasis in the future, as studies have demonstrated the essential functions of TEDs in cell-to-cell signaling and organotropism.

## The significance of PMN in BC intervention

Since PMN plays a critical role in BC metastasis, regulating and targeting PMN may provide promising strategies for early detection, treatment, and prognosis of BC metastasis.

### Identify PMN for early diagnosis of BC metastasis

Currently, there are no effective techniques for detecting cancer metastasis in the early stages. Although computed tomography (CT) and positron emission computed tomography (PET) is commonly used to identify cancer metastasis, their ability to detect micrometastases is limited 111, 112. Finding a tiny number of cancer cells in secondary organs at the start of metastasis is challenging, but their presence is critical for future therapy methods and cancer prognosis. Before cancer metastasis, the PMN forms, and many abnormal activities occur, which can be used to early diagnose cancer metastasis. For example, in lung metastasis, neutrophils are recruited to the lung tissues during BC lung spread, and overexpressed oxidative mediators are also observed 7, 113. Zheng and his coworkers designed luminous nanoparticles (LAD NPs) made of biocompatible, neutrophil-responsive self-illuminating cyclodextrin material and aggregation-induced emission agents, that use pulmonary infiltrated neutrophils as targets to detect BC lung metastasis. The results of the 4T1 BC lung metastasis of mouse model revealed a correlation between neutrophils and lung metastasis, as well as confirming the effects of LAD NPs in an early image of the PMN and detection of BC lung metastasis. Furthermore, the good safety and the detecting superiority of the targeting nanoprobe-based luminescence imaging strategy show great potential in utilizing the PMN to reveal BC metastasis 114.

Additionally, examining the components and secreted factors of PMN, such as TDEs may be used as potential biomarkers for detecting early BC metastasis. Lipid biopsy employs a similar principle to assess early cancer metastasis and progression 115. For instance, aberrant miRNA expression produced by BC-derived exosomes could regulate the target cells and contribute to organotropism metastasis. The use of exosomal miRNAs as blood-based biomarkers may open up new possibilities for predicting BC metastasis 116. Wang et al. showed that lower expression of miR-365-5p in the plasma exosomes is more readily investigated in BC patients with lymph node-positive, implying that miR-363-5p could serve as a candidate biomarker for BC lymph node metastasis 117. Higher expression of miR-21 indicates organotropism in BC bone metastasis predicts this 109. In addition to miRNA, the specific expression of cancer markers CD24, survivin, focal adhesion kinase (FAK), and epidermal growth factor receptor (EGFR) in BC-derived exosomes may be considered biomarkers for BC metastasis 118.

Although few researchers are focusing on using PMN to detect cancer metastasis early, these studies may provide different perspectives on the applications of PMN and approach to discovering early BC metastasis. As previously stated, numerous signaling pathways in the PMN are unusually activated or reduced. Several biological processes and cellular reactions are changed including immune cell recruitment, aberrant ECM remodeling, and overexpression of certain molecules. It is conceivable that novel biomarkers for tracking PMN formation and predicting metastasis will be discovered. Specifically, targeting or detecting these pathways and molecular and cellular components of PMN may provide novel concepts in the early diagnosis of BC metastasis, but there are many issues to consider, such as safety, efficacy, and specificity.

### The main strategies for targeting PMNs in BC metastasis treatment

**Figure 4:**
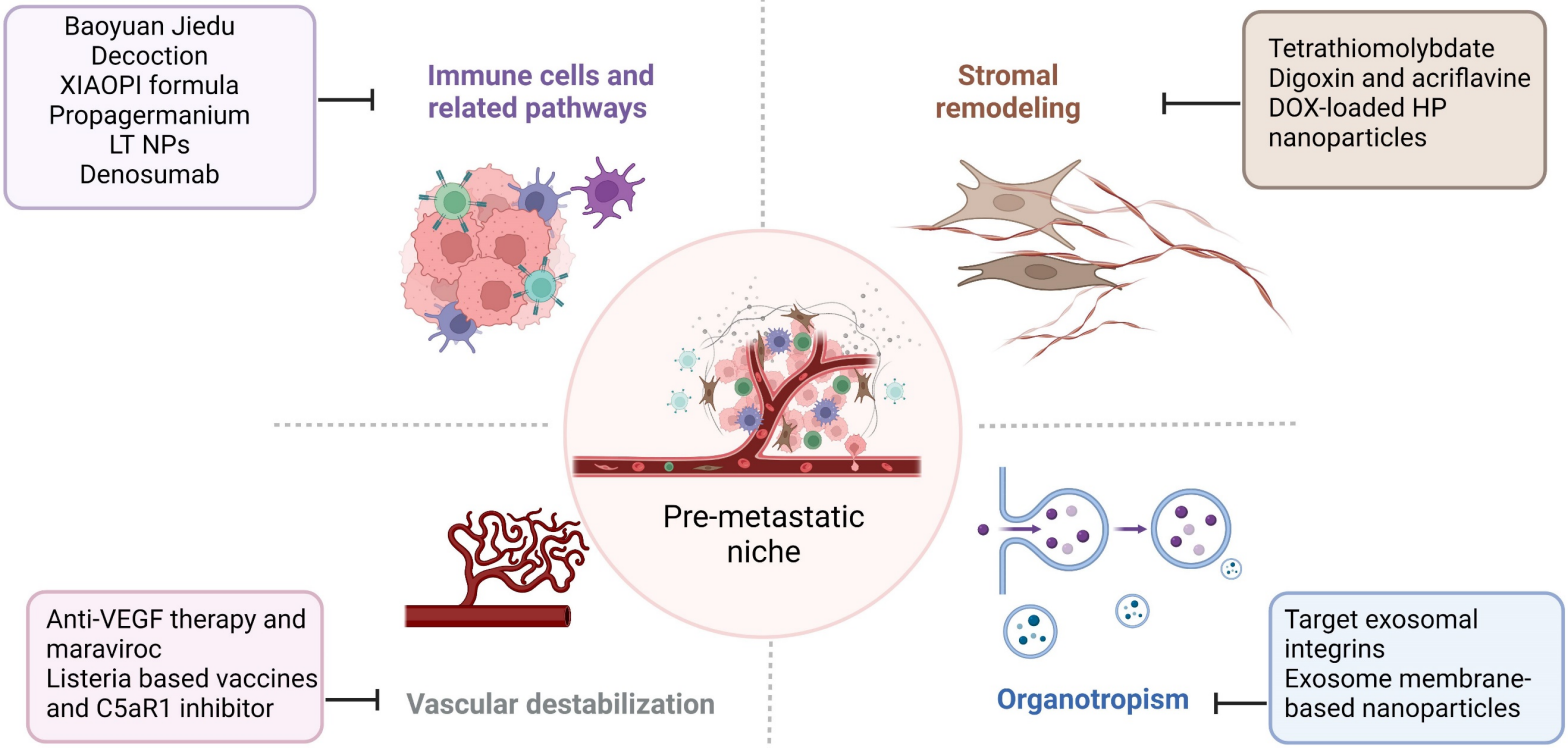
** Therapeutic strategies targeting the PMN in BC.** Several therapeutic options, such as targeting signaling pathways mediated by immune cells, stromal remodeling, vascular destabilization, and targeting organotropism, may prevent PMN formation and further metastasis in BC. The figure was created using Biorender (https://biorender.com/).

Tumor metastasis is challenging to treat, and the prognosis is poor. Given the importance of PMN in cancer metastasis, addressing the components and signaling pathways involved in PMN development and growth may provide successful therapeutic options for treating cancer metastasis. We focused on immune cells, stromal remodeling, vascular destabilization, and organotropism **(Figure 4).** We also identify known therapeutic reagents targeting PMN to inhibit BC metastasis (**Table 1**), which may encourage future possibilities in this area.

#### Target signaling pathways mediated by immune cells

Recruitment of BMSCs, such as MDSCs, can increase the likelihood of BC metastasis, which can promote PMN formation and generate the immunosuppressive microenvironment 20. Therefore, targeting the components or mechanisms that cause this phenomenon may be a viable means of preventing BC metastasis. TGF-β as an essential tumor regulatory factor may promote PMN formation by attracting MDSCs to target organs 119. Tian et al. showed that Chinese herbal medicine *Baoyuan Jiedu* decoction could inhibit the lung PMN formation and the recruitment of MDSCs in the lungs by suppressing the TGF-β/CCL9 signaling pathway, which promotes the directional movement of tumor cells and completes the lung-targeted metastasis 120. Another type of Traditional Chinese Medicine, the XIAOPI formula, could suppress BC lung metastasis by inhibiting CXCL1, which is secreted by TAMs. XIAOPI formula reduces the activation of hematopoietic stem/progenitor cells and their differentiation into MDSCs to prevent the formation of BC PMNs by suppressing the TAMs/CXCL1 signaling, indicating the potential of TAMs/CXCL1 as a therapeutic target in BC metastasis 121. CCL2, which binds to CCR2 and recruits MDSCs, could also be an attractive target. Propagermanium, a CCR2 antagonist may prevent cancer metastasis, and phase 1 dose-escalation trials showed its safety as an anti-metastatic drug for BC patients 122, 123. Adjuvant epigenetic therapy may be used, such as using 5-Azacytidine and entinostat, which are low-dose DNA methyltransferase and histone deacetylase inhibitors. These drugs can inhibit the movement of monocytic and granulocytic MDSCs, thereby preventing PMN formation by downregulating the CCR2 and CXCR2, 124. Such epigenetic therapy has been shown to have therapeutic effects in BC patients 125. Furthermore, with the advancement of nanomedicine, this approach could be used in anti-metastasis treatment. Long et al. developed LT NPs, self-delivery micelle nanoparticles with a low molecular weight heparin-tocopherol succinate (LMWH-TOS) that could bind strongly to the lung vascular endothelial cells (VECs) due to the adherence effects of LMWH and P-selectin. Therefore, the extravasation of granulocyte-MDSCs (G-MDSCs) regulated by the adherence of P-selection is inhibited, which reduces the accumulation of G-MDSCs and inhibits the formation of lung PMN in BC 21, 23.

RANKL is derived from tumor-specific T cells and has the potential to create an immunosuppressive bone PMN by regulating osteoclastogenesis to promote BC bone metastasis 31. Thus, targeting the RANK/RANKL signaling may be attractive for treating BC bone metastasis. A clinical trial found that using denosumab, a human monoclonal antibody targeting the RANKL, could reduce fractures and increase the prognosis of BC patients who are postmenopausal hormone receptor-positive and taking aromatase inhibitors 126. However, the most recent clinical study found that denosumab has no significant effects on the prognosis of patients with high-risk early BC 127. Although the therapeutic benefits of denosumab in BC bone metastasis are debatable, it suggests that targeting the RANK/RANKL signaling pathway may influence bone PMN formation and change the outcomes of BC bone metastasis.

#### Target stromal remodeling

As previously demonstrated, one of the most important features of the PMN is stromal remodeling. Targeting the components involved in this process may inhibit PMN progression and BC metastasis. According to Liu et al. a copper-depleting compound named tetrathiomolybdate can reduce the deposition of collagen, lower the accumulation of MDSCs, and boost the CD4+ T-cell infiltration, which alters collagen remodeling in the PMN and inhibits BC metastasis 128. A phase II clinical trial of patients with high-risk, triple-negative breast cancer verified therapeutic survival advantages 129. LOX is an enzyme involved in the cross-linking of collagen, which is one of the essential ECM components. Increased levels of LOX are being studied in the case of induced hypoxia, which increases the deposition of collagen and the stiffness of ECM to facilitate cancer metastasis 130-132. Therefore, LOX could be developed as a treatment target for BC metastasis. Wong et al. demonstrated that digoxin and acriflavine, as HIF-1 inhibitors, could suppress hypoxia-induced LOX proteins expression, and inhibit collagen cross-linking and the accumulation of BMDC, thereby preventing BC lung metastasis 133. Furthermore, the suppression of LOX reduces collagen crosslinking and this leads to a decline in the insoluble fibrotic matrix, resulting in the prevention of BC metastasis 132. Although the effects of targeting LOX proteins in BC metastasis have been shown in the above research, more literature and clinical trials are needed to understand the mechanism of LOX and evaluate the outcomes in BC metastasis. Additionally, Xiong et al. demonstrated that DOX-loaded hydroxyapatite (HP) nanoparticles modified with a high-affinity peptide for FB-FN called CREKA (Cys-Arg-Glu-Lys-Ala) peptide could bind and damage the primary tumor and PMN in 4T1 lung metastasis model, thus inhibiting the BC metastasis 134, 135. Overall, the concept of targeting stromal remodeling as a therapeutic strategy for BC metastasis has a long way to go.

#### Target vascular destabilization

Vascular instability is vital for BC metastasis because it affects PMN vascular permeability and angiogenesis. Anti-angiogenic therapies such as anti-VEGF therapy have been shown to have positive effects with maraviroc (CCR5 antagonist), an antiretroviral drug, to treat metastatic BC 97. Furthermore, studies have shown that when combined with antiangiogenic Listeria-based vaccines and inhibition of C5aR1, it is possible to reduce vascular density and boost antitumor immunity in the lung PMN, thereby preventing BC metastasis 136. Moreover, they found that this therapy outperformed sunitinib, a receptor tyrosine kinase inhibitor that interacts with PDGFR and VEGFR to elicit antiangiogenic functions 136.

#### Target organotropism

Exosomes play an important role in mediating organotropism in BC metastasis. For example, studies have revealed that BC-derived exosomes can particularly target lung tissues due to interactions between integrins on exosomes and laminin, which is abundant in the lung microenvironment 79, 137, 138. Therefore, targeting exosomes could be a promising strategy for preventing organotropism of BC metastasis. Exosomal integrins α6β4 and αvβ5 have been shown to prevent exosomes uptake by resident cells hence inhibiting further lung and liver metastasis 79. Nanomedicines could be extremely useful in this area. Ye et al. showed that nanosponges and nanokillers, gold nanocages modified with the platelet, and neutrophil hybrid cell membrane, could bind to high-affinity membrane adhesion receptors to remove interactions between tumor-derived exosomes and immune cells, which capture and eliminate circulating tumor cells and exosomes 139. Additionally, exosome membrane packaged core-shell nanoparticles may deliver drugs specifically to the organotropic PMN, which are useful in preventing cancer progression and effective in cancer treatment. Zhao et al. demonstrated that therapeutic siS100A4 could be transported by exosome membrane-based nanoparticles to the lung PMN using the principle of exosome organotropism, thereby preventing the degradation of siRNA and elucidating useful effects in cancer treatment 140.

## Perspectives and Conclusions

It is known that the formation of PMN is caused by various complex and dynamic cell-to-cell and cell-to-environment interactions. With the advancement of biological technologies, more cellular and molecular components and signaling pathways related to PMN have been revealed. However, many issues remain to be considered and further investigated, such as the specific inductive events in the PMN formation, the relationships between the primary tumor microenvironment and PMN in initiating the metastasis process, and the presence or absence of heterogeneity of PMN in different populations.

Although the clinical application of PMN in cancer is still in its nascent stages, we think it will become a viable method in the future assessment, prevention, and treatment of cancer metastasis. First, the advancement of exosome analysis technologies such as nano-plasmonic sensors and microfluidic exosome analysis opens the door to liquid biopsies, which can provide timely information about diagnosis and prognosis in cancer metastasis. Second, the rapid advancement of nanomedicines makes targeted therapies more effective and specific, which may aid in the development of new strategies to target PMN and apply the concept of precision medicine by providing individualized treatment based on individual differences. However, translating basic studies on PMN mechanisms into clinical practice is certainly challenging. Many issues, such as individual heterogeneity, complex network interactions between targets and other signaling pathways, adverse reactions, and efficacy need to be properly addressed.

To conclude, in BC metastasis, PMN may form in secondary organs through several mechanisms, including regulation of immune cells, stromal remodeling, angiogenesis/vascular permeability, metabolic reprogramming, and organotropism, all of which promote the tumor cells colonization and BC organotropic metastasis. Investigating the mechanisms of PMN formation may yield corresponding strategies for early diagnosis and treatment of BC metastasis.

## Figures and Tables

**Table 1:** Therapeutic strategies against PMN in BC clinic treatment

Therapeutic agent	Description	Outcomes	Citation
Baoyuan Jiedu decoction	Chinese herbal medicine	Suppress the TGF-β/CCL9 signaling pathway, inhibit the recruitment of MDSCs and lung PMN formation	120
XIAOPI formula	Chinese herbal medicine	Suppress the TAMs/CXCL1 signaling, decreases the activation of hematopoietic stem/progenitor cells and their differentiation into MDSCs	121
Propagermanium	CCR2 antagonist	Binds to CCR2 and inhibit the recruitment of MDSCs	122, 123
5azacytidine and entinostat	low-dose DNA methyltransferase and histone deacetylase inhibitors	Downregulate the CCR2 and CXCR2 to inhibit the movement of monocytic and granulocytic MDSCs	124
LT NPs	LMWH-TOS self-delivery micelle nanoparticle	Bind to the VECs, block the extravasation of VECs, and decrease the accumulation of G-MDSCs	21, 23
Denosumab	Anti-RANKL	Target the RANK/RANKL signaling	126
Tetrathiomolybdate	A copper-depleting compound	Reduce the deposition of collagens, decrease the accumulation of MDSCs, and increase the CD4+ T-cell infiltration	128, 129
Digoxin and Acriflavine	HIF 1 inhibitor	Suppress the expression of LOX proteins induced by hypoxia, inhibit collagen cross-linking and the accumulation of BMDC	133

## Abbreviations

BC: breast cancer
PMN: pre-metastatic niche
TDSFs: tumor-derived secreted factors
BMDCs: bone marrow-derived cells
EVs: extracellular vesicles
HIF-1α: hypoxia-inducible factor-1α
TGF-β: transforming growth factor-β
MDSCs: myeloid-derived suppressor cells
VEGF: vascular endothelial growth factor
VEGFR-1: vascular endothelial growth factor receptor-1
PTEN: phosphatase and Tensin homolog
AKT: protein kinase B
NF-κB: nuclear factor kappa-B
CCR: C-C chemokine receptor
CXCL: chemokine (C-X-C motif) ligand
CXCR: chemokine (C-X-C motif) receptor
APCs: antigen-presenting cells
DCs: dendritic cells
RANKL: receptor activator of the nuclear factor kappa B ligand
OCs: osteoclasts
BM: bone marrow
TIL-Bs: tumor-infiltrating B cells
LNs: lymph nodes
GC: germinal centers
Tfr: T follicular regulatory cells
Tregs: regulatory T cells
TAMs: tumor-associated macrophages
CYP: cytochrome P450
STAT3: signal transducer and activator of transcription 3
PD-L1: programmed cell death-ligand 1
MSCs: mesenchymal stem cells
ECM: extracellular matrix
CAFs: cancer-associated fibroblasts
HGF: hepatocyte growth factor
MIF: macrophage migration inhibitory factor
OPG: osteoprotegerin
NETs: neutrophil extracellular traps
TF: tissue factor
PAR-1: protease-activated receptor 1
HA: hyaluronan
FN: Fibronectin
TNC: Tenascin-C
HPCs: hematopoietic progenitor cells
DTC: disseminated tumor cells
LOX: lysyl oxidase
PGE2: prostaglandin E2
LECs: lymphatic endothelial cells
TDEs: tumor-derived exosomes
LAD NPs: luminescent nanoparticles
FAK: focal adhesion kinase
EGFR: epidermal growth factor receptor
LMWH-TOS: low molecular weight heparintocopherol succinate
CTCs: circulating tumor cells
TLS: tertiary lymphoid structures
BoMAMs: bone metastasis-associated macrophages
TNBC: triple-negative breast cancer
CHI3L1: chitinase 3-like 1
CECR2: cat eye syndrome chromosome region candidate 2
RELA: v-rel avian reticuloendotheliosis viral oncogene homolog A
COX-2: cyclooxygenase-2
IL: interleukin
CTSC: tumor-secreted protease cathepsin C
PR3: proteinase 3
